# Obesity as a Risk Factor for Breast Cancer—The Role of miRNA

**DOI:** 10.3390/ijms232415683

**Published:** 2022-12-10

**Authors:** Karolina Hanusek, Jakub Karczmarski, Anna Litwiniuk, Katarzyna Urbańska, Filip Ambrozkiewicz, Andrzej Kwiatkowski, Lidia Martyńska, Anita Domańska, Wojciech Bik, Agnieszka Paziewska

**Affiliations:** 1 Department of Biochemistry and Molecular Biology, Centre of Postgraduate Medical Education, ul. Marymoncka 99/103, 01-813 Warsaw, Poland; 2 Department of Neuroendocrinology, Centre of Postgraduate Medical Education, Marymoncka 99/103, 01-813 Warsaw, Poland; 3 Department of General, Oncological, Metabolic and Thoracic Surgery, Military Institute of Medicine, 128 Szaserów St, 04-141 Warsaw, Poland; 4 Laboratory of Translational Cancer Genomics, Biomedical Center, Faculty of Medicine in Pilsen, Charles University, Alej Svobody 1665/76, 32300 Pilsen, Czech Republic; 5 Faculty of Medical and Health Sciences, Institute of Health Sciences, Siedlce University of Natural Sciences and Humanities, 08-110 Siedlce, Poland

**Keywords:** breast cancer, obesity, inflammation, estrogens, miRNA, hypoxia, metabolites

## Abstract

Breast cancer (BC) is the most common cancer diagnosed among women in the world, with an ever-increasing incidence rate. Due to the dynamic increase in the occurrence of risk factors, including obesity and related metabolic disorders, the search for new regulatory mechanisms is necessary. This will help a complete understanding of the pathogenesis of breast cancer. The review presents the mechanisms of obesity as a factor that increases the risk of developing breast cancer and that even initiates the cancer process in the female population. The mechanisms presented in the paper relate to the inflammatory process resulting from current or progressive obesity leading to cell metabolism disorders and disturbed hormonal metabolism. All these processes are widely regulated by the action of microRNAs (miRNAs), which may constitute potential biomarkers influencing the pathogenesis of breast cancer and may be a promising target of anti-cancer therapies.

## 1. Introduction

### 1.1. Epidemiology

Breast cancer (BC) is the most common cancer among women worldwide [1,2]. Approximately 0.5–1% of breast cancers are diagnosed in men. WHO reports that breast cancer has the highest cancer incidence rate (11.7%, 2,261,419 cases in 2020) and a significant increase in mortality (6.9%, 684,996 deaths in 2020) [3,4]. Despite the development of new medical technologies in medicine and the progress in diagnostics and therapy, breast cancer is still a big challenge. It is estimated that breast cancer will develop in one in five people worldwide during their lifetime. Unfortunately, the forecasted statistics suggest that these numbers will increase and in 2040 will be nearly 50% higher than in 2020 [5].

**Figure 1:**
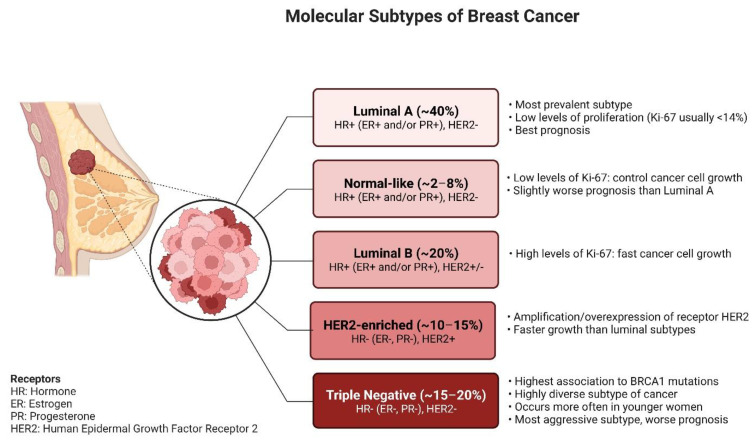
Classification of breast cancer. The above figure was created with BioRender.com. Agreement number: *MV24QP1OWO*.

Breast cancer classification is based on cytological, immunohistochemistry, and molecular patterns. Invasive ductal carcinoma and invasive lobular carcinoma, which are the most common subtypes, represent approximately 90% of breast cancer cases. Immunopathological methods describe types of breast cancer by the presence or absence of receptors, and additionally, molecular biology methods allow categorization further into subtypes based on mRNA expression [6,7,8]. Subtypes of breast cancer are classified by the presence of hormone receptors (including estrogen ER and progesterone PR receptors), human epidermal growth factor receptor 2 (Her2 status) as based on a subdivision on luminal A and luminal B, and triple-negative (ER-, PR-, HER2-) [9,10,11,12,13] [Figure 1]. Histochemical staining for the proliferation marker protein Ki-67 should be mentioned at this point as it may be used to differentiate between luminal A and luminal B breast cancer types [9]. All these subtypes constitute a diverse group of tumors characterized by different pathogenesis, treatment needs, responses to a particular treatment, and disease-specific outcomes [14].

Breast cancer is a heterogeneous disease with phenotype variations. Tumor heterogeneity may be observed between patients—intertumoral heterogeneity, and inside a single tumor—intratumor heterogeneity [15,16]. Inherited or de novo mutations of genes are responsible for cancer initiation and are additionally important elements of breast cancer classification.

Here, we present obesity as a growing risk factor for breast cancer development. This study aims not only to discuss the inflammation process, the metabolic and hormonal disturbances related to obesity analyses in the female population but also, mainly, the regulatory mechanism of microRNAs. Participation and the role of specific individual microRNAs in inflammation, disrupted metabolism of glucose, lipids, and hormones in obesity-related cancer could be used as potential biomarkers in the pathogenesis of breast cancer or be a promising target of anti-cancer therapies.

### 1.2. Pathogenesis of Breast Cancer

Breast cancer is a malignant disease that is initiated in the cells of the breast and creates its specific tumor microenvironment (TME). Clinical presentation of breast cancer may depend on various factors, such as location, type of tumor, or stage of the disease. A palpable lump or bump, size change, replacement of the nipple, pain, skin change, ulceration, and even swelling of the limb are the most frequently mentioned symptoms of advanced breast cancer. However, even when there is no signs, symptoms, or palpable mass, the disease may develop, so prevention and control checks and tests should not be underestimated.

Many causes and risk factors lead to tumor development. Family history and inheritance of mutations of one of the most significant and common genes, BRCA1, BRCA2, or at least seven other genes, ATM, CDH1, CHEK2, NF1, PALB2, PTEN, and TP53, are proven risk factors for breast cancer initiation and development [17,18]. Among the environmental risk factors are obesity related to disrupted metabolism, higher production of prooncogenic estrogen, and chronic inflammation [19], which constitute the background of the oncogenesis of breast cancer. These issues will be presented in our review.

Obesity is defined as the higher amount and distribution of unhealthy fat mass [20,21]. An indirect indicator of body fat is a body mass index (BMI) greater than or equal to 30, defined as a person’s weight in kilograms divided by the square of his height in meters (kg/m^2^) [22,23]. Another useful index is the waist–hip ratio (WHR). Since 1975, the number of obese people has tripled almost three times, according to the World Report (WHO). In 2016, more than 1.9 billion adults, 18 years of age and older, were overweight [22] and this rate is still rising rapidly (52.7% of adults in 2019) in most of the EU’s population. As reports show, obesity is a growing problem all over the world [22,24,25]. On the one hand, it is the disease itself, and on the other hand, it can lead to the development and progression of obesity-related cancers, including breast cancer, which is significantly associated with BMI and fat mass. Increasing evidence has indicated the association between obesity cases and the number of newly diagnosed patients with breast cancer [26,27]. Approximately 7% of all postmenopausal (over 50 years old) breast cancer cases (114,800 cases) in 2012 were associated with excess body weight. With the increase in BMI, the risk of developing breast cancer after menopause increases and amounts to 12% for every five-point increase in BMI [28]. The incidence of breast cancer is also higher in patients diagnosed with diabetes. Additionally, clinical observations show that obesity-related hyperglycemia causes faster and more aggressive progression of oncological diseases and reduces overall and cancer-free survival [29,30,31,32].

Obesity patients are diagnosed with larger breast cancer primary tumors and advanced disease stage at the time of diagnosis [33]. Furthermore, obesity-related inflammation promotes not only the initiation but also the progression of breast cancer along with angiogenesis observed in the mammary tissue of the majority of obese and diabetic individuals [27]. Up to 46% more distant metastases 10 years after diagnosis are diagnosed in a population of breast cancer obese patients (with a BMI of 30 kg/m^2^) or more) compared with patients with a BMI below 25 kg/m^2^ [34]. The risk of developing lymph node metastases, increases additionally for every 1 kg/m^2^ increment of BMI, regardless of the premenopausal or postmenopausal status [33].

Adipose tissue (AT) is a complex disseminated endocrine organ consisting of adipocytes and fibroblasts, adipocytic progenitor cells, and immune cells: lymphocytes B, lymphocytes T, and macrophages [35]. AT models and crosses are the key pathways of cellular metabolism [36]. The interplay between breast cancer and obesity is connected with metabolism disturbances associated with glucose metabolism (glycolysis, Warburg effect, and hypoxia), lipid metabolism (lipogenesis and lipolysis, and cholesterol metabolism), insulin resistance, disturbed signaling of insulin-like growth factor (IGF-1), and oxidative stress. Apart from the listed above factors, which cause oxidative stress, adipocyte hypertrophy and systemic inflammation should also be mentioned. The most important biomarkers of oxidative stress detected in obese patients are products of lipid peroxidation: isoprostanes, thiobarbituric reactive acid substances (TBARS), or malondialdehyde (MDA), and lipid hydroperoxides (LOOH) [37,38,39]. Prolonged exposure to ROS leads to DNA damages in breast epithelial cells and increases the risk of cancer initiation.

In obese individuals, differences in the number, size, and characteristics of adipocytes are observed. Adipocytes are the source of many hormones, named adipokines, such as leptin, adiponectin, resistin, visfatin, and apelin. Inhibition of apoptosis, angiogenesis, and promotion of cancer cell proliferation and migration may involve high levels of leptin, while low levels of adiponectin may interrupt the AMP-activated protein kinase (AMPK) and the insulin-signal pathways, which lead to cancer cell proliferation [40]. In detail, the following pathways and compounds are involved: extracellular signal-regulated kinase (ERK) signaling pathways, Janus kinase (JAK/STAT), AMPK, phosphatidylinositol 3-kinase (PI3K), and mitogen-activated protein kinase (MAPK) [41,42].

Adipocytes are responsible for the synthesis of most of the estrogens from androgens after menopause [43]. An elevated level of estrogen is one of the protumorigenic breast cancer risk factors for women with obesity. It increases the risk of developing the disease and influences the severity at the time of diagnosis. Additionally, obesity-related disturbances in aromatase expression responsible for estrogen production are observed during the initiation of cancer and linked to the progression of breast cancer [44,45]. Accumulation of adipose tissue is connected with chronic inflammation, which is also a hallmark of cancer, and together with disrupted metabolism may serve as significant risk factors for the development of breast cancer [46,47]. Additionally, adipocytokine pathophysiology, disturbed differentiation of macrophages, and secretion of inflammatory molecules (IL-6, IL-8, IL-5, IL-13, TNF-α) are reported [46]. Cytokines regulate and modulate the immune response and provide complex communication between cells and organs and are responsible for sustaining homeostasis. Macrophages present pro-inflammatory (M1) or anti-inflammatory (M2) phenotypes [48], and together with T-cells (CD4+ and CD8+ T-cells) and secreted inflammatory cytokines they are an essential component of the innate immune system.

Accumulation of macrophages causes a chronic inflammatory state and, consequently, leads to additional metabolic disturbances [49]. Due to obesity and secretion of selected cytokines (IL-6 and TNF- α), the anti-inflammatory phenotype of macrophages changes into a proinflammatory phenotype [50]. The percentage of M1 macrophages increases from 5% to 50% in AT of obese people compared to lean patients [51,52,53]. Additionally, primary tumors also secrete cytokines to recruit inflammatory response cells (e.g., monocytes, granulocytes) into the tumor and, consequently, promote chronic inflammation. These differentiated tumor-associated macrophages (TAMs) are responsible for tumor growth [53,54,55] and progression of cancer, and are, subsequently, strongly correlated with poor prognosis [56]. Notably, significantly higher TAMs infiltration is observed in the normal and the cancerous tissues of hyperglycemic patients. This inflammation process present in the tumor environment is associated with a poorer outcome in patients with breast cancer [57,58].

## 2. microRNAs (miRNAs) in Breast Cancer

### 2.1. miRNAs and Obesity

The complex organization of cells and tissue requires global control and regulation. Over the past few decades, it has been described that microRNAs (miRNAs) regulate many cellular processes, such as proliferation, differentiation, angiogenesis, and metabolism. Increasing data show that both adipose tissue and cancer cells produce specific miRNAs that have a profound effect on gene expression in other cells and tissues.

miRNAs are endogenous noncoding small RNAs consisting of 19–23 nucleotides. Non-coding miRNAs control many crucial physiological processes in the pathogenesis of various oncological diseases, including breast cancer [27]. Moreover, the identification of changes in miRNA expression profiles leads to the determination of miRNA signatures typical to different molecular breast cancer subtypes classified by receptors statuses: ERα+, Her2, and TNBC [59], and can be used as a potential diagnostic or prognostic biomarker [60]. Moreover, miRNAs can serve as novel targets for subtype-specific therapy [60].

miRNAs can function as tumor suppressors, and their reduced expression may be associated with the initiation of the neoplastic process. On the other hand, miRNAs can promote tumorigenesis and show increased expression levels in tumors associated with a specific tumor or type of histological pattern and could, therefore, be referred to as “oncomiR”.

**Figure 2:**
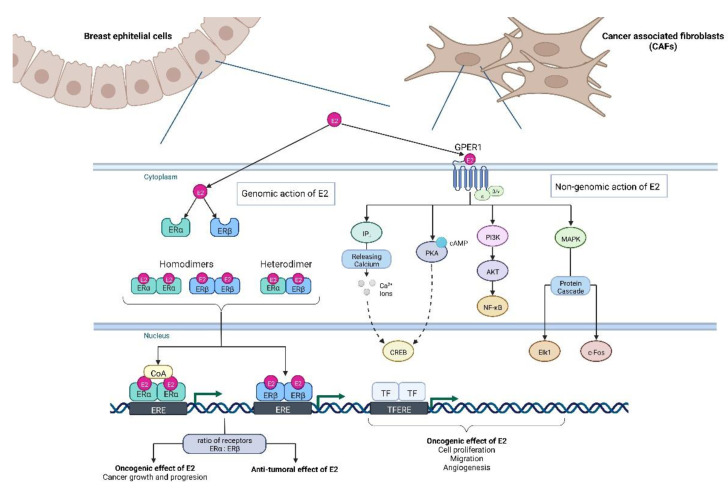
Estrogen Receptor signaling in breast epithelium cells and CAFs. Mechanisms of E2 action in cells can be divided into genomic action through binding to nuclear estrogen receptors ERα or Erβ, and non-genomic action by binding to GPER1 [131]. ERα and ERβ are ligand-activated transcription factors that mediate the effect of estrogens by binding with specific estrogen response elements (EREs) in promotors of target genes, which regulate the transcription of these genes [132]. ERα and Erβ, after activation by E2 binding, form homo- or heterodimers with each other. Activation of ERα promotes proliferation, tumor growth, and angiogenesis through the induction of expression of oncogenes like c-MYC, VEGF, or CCND1 [133,134,135]. The additional layer of the regulation of E2 genomic action is the interaction of ERs with various coactivators (CoA), such as transcription factor Sp1 (SP1), which modulate the binding of activated E2-ER complex with ERS [136]. Non-genomic action of E2 via binding to GPER1 leads to signal transduction through the G protein, which results in the synthesis of cAMP [137,138,139] and activation of downstream signaling pathways: phosphatidylinositol 3-kinase (PI3K), Akt kinase, Ca^2+^ signaling, and MAP kinases [140]. In breast tumors, estrogenic activation of GPER1 leads to the induction of proliferation, migration, or angiogenesis [141,142]. GPER1 is expressed also in breast cancer CAFs [143,144,145]. Activation of GPER1 signaling may lead to an increase in CAFs proliferation rate or migration by induction oncogenes expressions like c-Fos or Cyclin D1 (CCND1) [146]. Additionally, Lappano et al. demonstrated that estrogenic activation of GPER1 in CAFs promotes breast cancer development by increasing angiogenesis via activation of HIF1-α and VEGF expression [147]. The next mechanism, which leads to the promotion of breast cancer progression by CAFs via GPER1, is the induction of the expression of pro-inflammatory cytokine IL1B and its receptor ILR1 in breast cancer cells and CAFs [148]. The above figure was created with BioRender.com. Agreement number: TM24NOJWQS.

In adipose tissue, miRNAs regulate the critical signaling pathways related to adipogenesis, adipocyte lipid storage, metabolism, and secretion of adipokines by affecting the genes that encode proteins involved in signaling and transcriptional regulation [61] [Figure 2].

The effect of mature adipocytes (MA; differentiated murine 3T3-L1 cells line) that increase the invasiveness of breast cancer cells was examined on the modulation of global miRNAs expression in breast cancer cells (human breast metastatic adenocarcinoma MDA-MB-231 cells line) using indirect in vitro co-culture model system (MA-BCa cells) [62]. Through a genome-wide analysis, the authors identified 98 miRNAs that were differentially expressed in breast cancer cells, which were co-cultured with MA in comparison to breast cancer cells cultured alone. The most changed miRNAs were miR-3184-5p and miR-181c-3p. The inhibition of miR-3184-5p or overexpression of miR-181c-3p significantly decreased cell numbers and invasion of breast cancer cells after co-culture with MA. Moreover, authors described that concomitant use of miR-3184-5p inhibitor and miR-181c-3p mimic inhibited the adipocytes-stimulated proliferation and invasion of breast cancer cells. These effects were exerted by targeting: a forkhead box protein P4 (FOXP4)—a direct target of miR-3184-5p; and peroxisome proliferator-activated receptor alpha (PPARα)—a target for miR-181c-3p, which are known to regulate the growth and metastasis of cancer cells in different kinds of tumors. [62].

Published studies have indicated that upregulated miR-9-5p may also be implicated in the development of obesity in humans. miR-9-5p downregulated the 3′UTR of Wnt3a (a Wnt ligand), which resulted in inhibition of Wnt/β-catenin signaling and stimulation of differentiation of rat marrow stromal stem cells (MSCs) into adipocytes [63]. Additionally, it was observed that miR-9-5p upregulated adipogenesis-associated genes, such as adipsin, peroxisome-proliferator-activated receptor γ (PPARγ), and CCAAT/enhancer-binding protein α (C/EBPα) in MSC cells [63]. Importantly, using a combination of bioinformatics analysis and luciferase activity assay, it was shown that miR-9-5p negatively regulated antitumoral adiponectin (ADIPOQ) [64]. In vitro studies indicated that adiponectin treatment increased autophagy in breast cancer cells (MDA-MB-231 and MCF7 cells) [64]. Additionally, the resistance of breast cancer cells to the treatment agent, tamoxifen, may be facilitated by an exosomal transfer of miR-9-5p and negative regulation of ADIPOQ [65]. Interestingly, high expression of adiponectin, adiponectin receptor 2 (ADIPOR2), and beclin 1 (BECN1, the protein implicated in the autophagic programmed cell death), significantly correlated with increased overall survival in chemotherapy-treated breast cancer patients [64].

A similar effect associated with upregulation of miR-221-3p and miR-222-3p was observed in the white adipose tissue of C57BL/6 mice fed with high high-fat sucrose chow [66]. These observations indicated that miR-222 could promote the proliferation of preadipocytes and the accumulation of lipids in mature adipocytes by inhibiting lipolysis [67] and, therefore, the overexpression of miR-222 is linked to enhanced adipogenesis. These observations indicate that miR-222 could be a potential candidate for miRNA-based therapy. miR-221-3p is not only related to metabolic diseases but is also considered a cancer-associated miRNA. miR-221-3p is abundantly expressed not only in tumor tissues but also in the circulation of patients with different types of cancer. Studies confirmed a cross-talk between miR-221-3p overexpressing adipocytes and MCF-7 BC cells. Moreover, miR-221-3p, by targeting different adipogenic genes (such as glucose transporter 4 (GLUT4), stearoyl-CoA desaturase-1 (SCD1), diacyl glycerol acyl transferase1/2 (DGAT1/2), fatty acid synthase (FAS), adiponectin (AdipoQ), adipocyte triglyceride lipase (ATGL), and adipocyte fatty acid binding protein (AP2)), inhibited the adipocyte differentiation process [61].

Secondly, miR-221-3p targeted 14-3-3γ and played a crucial role in the repression of adipocyte differentiation. The protein 14-3-3γ belongs to a family of small, acidic 14-3-3 proteins that, through functioning adaptors, scaffolds, and chaperones, regulate various cellular functions, such as cell growth, survival, differentiation, migration, and other intracellular signal transduction pathways [68]. Importantly, the elevated expression of miR-221-3p was observed in the breast adipose tissue, which was isolated from patients with grade III invasive breast cancer [61]. Moreover, in invasive breast cancer, the adiponectin expression in the adipose tissue decreased and negatively correlated with miR-221-3p, which indicated disturbed adipogenesis near the breast cancer tumor [61]. Finally, the published results showed that the conditioned medium obtained from miR-221-3p overexpressing adipocytes induced the proliferation and invasion of MCF-7 cells. However, the medium obtained from MCF-7 cells culture increased expression of miR-221-3p in adipocytes. The authors assume that changes in the tumor microenvironment, such as reduced adipogenesis and disturbances in adipokine expression, could have facilitated the proliferation and migration of cancer cells [61].

These results are of high interest because they indicate cross-talk between cancer cells and the nearby adipocytes, which could stimulate the progression of tumors [61].

Mature adipocytes seemed to upregulate their beige/brown depot and undergo a lipolytic process, with a release of metabolites such as pyruvate, free fatty acids, ketones, and lactate after co-culture with human breast cancer cell lines (MCF-7, MDA-MB-231, and HEK 293 T) [69].

However, tumor cells that were co-cultured with mature adipocytes presented metabolic adjustments and an aggressive phenotype. In this context, miR144 and miR126 play an important role in altering energy metabolism, which in turn facilitates tumor progression. Downregulate activity of the Mitogen-Activated Protein Kinase Kinase Kinase 8/Mitogen-Activated Protein Kinase 3/1/PPARγ axes (MAP3K8/ERK1/2/PPARγ) by miR144 induced brown/beige-like characteristics in differentiated adipocytes. Exosomal miRNA-126 affected metabolism by affecting other signaling pathways. In detail, miR126 targeted insulin receptor substrate 1 (IRS1), which in turn activated the Protein Kinase AMP-Activated Catalytic Subunit Alpha 1 (phosphor-AMPKα)/autophagy pathway and stabilized expression of HIF1α in adipocytes [69].

Another oncogenic miRNA, miR-155, is involved in insulin resistance and was found in the exosomes secreted from adipose tissue macrophages (ATM) isolated from human individuals with obesity [70] as well as secreted by the ATM in the murine model [71]. Moreover, an increased expression of miR-155 was found in ATM isolated from human individuals with obesity and in a murine macrophage RAW264.7 cell line [71]. In recent years, both in vitro and clinical studies have shown that miR-155 played an important role in breast cancer [72,73] and significantly enhanced the proliferation and migration of the breast cancer cell line MBA-MD-231 [72]. Furthermore, upregulation in the relative expression of plasma miR-155 was observed in early-stage breast cancer patients [73]. Based on the above data, the authors suggested that miR-155 might serve as an oncogene in breast cancer [72].

miRNA can influence metabolism as vital regulators of energy metabolism (e.g., glucose metabolism, lipid metabolism, and amino acid biogenesis) or target metabolic factors that modulate their expression [74]. It is a well-known fact that cancer cells exhibit altered metabolism, which plays a pivotal role in cell transformation, cancer initiation, and progression [75]. Cancer cells increase glucose uptake and alter glucose-related pathways (e.g., glycolysis, PPP). GLUTs (glucose transporters) are responsible for glucose transport through the plasma membrane into the cytoplasm [76]. GLUT1 expression is connected with poor prognosis in breast cancer [77]. Moreover, it was proven that its expression is inversely correlated with miR-22. Thus, these factors interplay shorter disease-free and overall survival [78]. A different example of regulation by miRNA can be exosomal miR-122, whose levels correlate with the metastatic capacity of breast cancer and inhibits glucose uptake in non-tumor cells, downregulating PKM2 and enhancing proliferation [79]. Another source of energy and the main energetic pathway for cancer cells is glycolysis. There is ample evidence that miRNA can control key enzymes of this pathway. The last glycolytic enzyme LDHA is a direct target for miR-34a, and both are negatively correlated in breast cancer. Furthermore, miR-34a can inhibit LDHA-induced glycolysis [80]. Similarly, miR-30a-5p can inhibit LDHA expression, which reduces glucose uptake, lactate production, and ATP generation [81,82].

Glutamine fuels the TCA cycle and is a vital source of carbon and nitrogen for nucleotide biosynthesis [83]. It was shown that epigenetic downregulation of miR-137 in cancer cells resulted in increased glutamine uptake and cancer cell survival in an unfavorable environment [84]. In breast cancer cells (MCF-7), miR-22 could lower invasiveness by inhibiting ACLY expression [85]. Interestingly, extracellular vesicles (EVs) can also affect glutamine metabolism. It was shown that MDA-MB-231 triple-negative cancer cells could release EVs with miR-105, which influence CAFs to regulate c-Myc. That leads to CAF metabolism modification and an increase in glutaminolysis and glycolysis [86].

Recently, more evidence showed miRNA-dependent metabolism modification not only in energy metabolism but also in a plethora of other metabolic processes with an impact on a patient’s survival, prognostication, and therapy. For example, levels of tryptophan-2,3-dioxygenase (TDO2)-rate-limiting enzyme for tryptophan catabolism are induced in triple-negative breast cancer [87]. Tryptophan catabolism plays a vital role in tumor progression [88]. It was demonstrated that miR-200c targeted TDO2 directly, which led to reduced production of Kynurenine, an immune-suppressive metabolite [89]. miR-125b promoted migration and invasion of MCF-7 cells and played a role in chemotherapy resistance [90,91]. It was proved that calcitriol, an active metabolite of Vitamin D3, and tacalcitol, a synthetic analog of vitamin D3, can decrease miR-125b expression and, in addition, increase the level of BAK1 pro-apoptotic protein [92].

On the other hand, some metabolites can also modulate levels of miRNA. For example, fatty acids (linoleic acid (LA) and docosahexaenoic acid (DHA)) can modulate breast cancer cells [93]. They can act independently or in a combined fashion. Some studies showed that they can induce apoptosis and reduce the invasiveness of breast cancer cells; however, other studies are reporting adverse results [94,95,96]. Interestingly, cells treated with LA showed changes in miRNA expression profiles (increased expression of miR-30 and miR-106b and decreased expression of miR-20, miR-126, and miR-194) [97]. These miRNAs play important roles in different processes: miR-20 promotes angiogenesis and the progression in breast cancer [98], miR-30 may suppress the progression of tumor cells in breast cancer [99], and miR-106b correlates with tumor size and metastasis [100]. It was shown that LA or DHA can decrease miR-20 expression and a combination of LA and DHA can induce miR-106b expression [97].

Significantly, excessive growth of adipose tissue due to obesity causes local hypoxia. Hypoxia is indicated as a microenvironmental factor that activates inflammation, deregulates homeostasis, and, consequently, results in cancer development [27]. Initiation of cancer, followed by uncontrolled proliferation of the tumor cells, and rapid growth of the tumor with the inefficient supply of blood to cells, results additionally in limitation of oxygen from 2–9% to the state of hypoxia (less than 2%). Hypoxia accompanying the cancer progression affects the expression of the genes, especially those related to metabolism and angiogenesis, like glucose intake or enzymes involved in glycolysis.

One of the most important factors of hypoxia is the hypoxia-inducible factor (HIF-1α), a transcription factor crucial for oxygen homeostasis and energy metabolism, cell survival, and tumor invasion. HIF-1α activates the expression of glucose transporters (GLUT-1, GLUT-3), vascular endothelial growth factor (VEGF), adrenomedullin (ADM), transforming growth factor-*β*3 (TGF-*β*3), hexokinase (HK1, HK2), insulin-like growth factor 2 (IGF2), IGF-factor-binding proteins (IGF-BP2, IGF-BP3), TGF-*α*, myelocytomatosis virus oncogene cellular homolog (C-MYC). It leads to modification of metabolism, which adapts neoplastic tissue to hypoxia, enables the survival of cells, and increases their further proliferation [27,101].

HIF-1 increases the expression of enzymes involved in glucose metabolism leading to a metabolic switch from oxidative metabolism to anaerobic glycolysis, which is named the Warburg effect. High glucose uptake can be observed in the presence of oxygen leading to the synthesis of lactate. An increased level of cellular glucose affects GLUT-1 expressions in cancers, which are associated with worse prognosis for patients [27].

Additionally, angiogenesis and metastasis are controlled through miR-373-TXNIP-HIF1a-TWIST signaling in breast cancer. Overexpression of miR-1275 activating Wnt/β-catenin is associated with poor prognosis linked to tumor progression metastasis, and recurrence [101]. Observed in obese patients, alternations of miRNA expression (for example miR-210 and miR-153) influence genes of the hypoxia pathways HIF1A, HK2, and VEGFA during invasion and proliferation of cell culture or tumor breast tissue [102,103,104].

### 2.2. miRNA and Inflammation

According to available data, miRNAs related to obesity are crucial components and regulators of signaling pathways linked to the functioning of adipocytes, monocytes, and other immune cells. The disrupted miRNA profiles are connected with inflammation resulting from obesity and with the tumor microenvironment [46]. Various inflammatory genes are activated and expressed to induce a pro-inflammatory immune response and additionally correlate with metabolic syndrome. miRNAs as suppressors or oncogenes influence inflammatory molecules and regulate the secretion of cytokines (interleukins, TNF-α, transforming growth factor-β (TGFβ), granulocyte-macrophage colony-stimulating factor (GM-CSF), chemokines transcription factors (nuclear factor kB (NF-kB), and signal transducer and activator of transcription 3 (STAT3)), and play a crucial role in the development of cancer-related inflammation and functioning of immune cells and immunity system.

Regulation with miRNAs as connected with obesity is not only related to metabolic pathways associated with obesity and inflammation, but also, additionally, to tumor initiation and progression (migration and metastasis: miR-200, miR-10b, miR-21, miR-494, and angiogenesis miR-16, miR-92a, and miR-21). The significant role of microRNAs is studied according to immunity and the inflammation accompanying breast cancer pathogenesis. Selected signaling pathways, MAPK, nuclear factor kappa B (NF-kB), Janus kinase/signal transducers and activators of transcription (JAK-STAT), toll-like receptor (TLR) pathways, cGAS/STING, and MAP kinase connected with chronic inflammation are supposed to be responsible for the development of cancers. They are related to the severity of the disease, the progression process, and even the response to treatment [105].

The tumor microenvironment is the complex of blood vessels, endothelial cells, cancer-associated fibroblasts (CAFs), stromal cells, and infiltrating immune cells with tumor-associated macrophages (TAMs) [106]. This complex influences the inflammation processes and the development of tumors [107,108]. Tumor cells communicate with inflammatory cells through cytokines (IL-1, IL- 6, IL-17, IL-23, and TNF-α), growth factors (TGF- β, colony-stimulating factors, and transcription factors), and miRNAs (miR-221 and miR-222). The interplay between these molecules, the adipose tissue, and breast endothelial cells is crucial in the development of the inflammatory microenvironment, and, consequently, increases the risk of cancer development. For example, exosomal miR-21 attaches with Toll-like receptor 7/8 (TLR7/8) on macrophages and helps to promote the secretion of IL-6, connected with the pro-inflammatory response. IL-6 stimulates STAT3 to activate the transcription of miR-21 [109] and miR-181-b1 [110,111].

miRNAs regulate the biogenesis and differentiation of adipose tissue. They are additionally released by adipocytes. Moreover, miRNAs may influence expression of miRNAs in adipocytes and inflammatory cells (e.g., T-cells, macrophages). Furthermore, miR-150 modulates AT function by B-cell activation and correlation with other immune cells. Expression of miRNAs is associated with the severity of obesity. miRNA expression profiles correlate not only with body mass index (BMI), waist-to-height ratio, and a percentage of fat mass but additionally with the inflammation process of dysplastic tissues. They are significantly different in both lean and obese individuals. Their important role is known in cancer development and progression [112,113,114].

Inflammation with deregulated cytokines IL-1, TNF, IL-6, and IL-23/IL17 is reported as a crucial factor for the development of cancer and is controlled and modified with miRNAs [115]. Discovered association between high levels of miR-21-5p of macrophages and expression of proinflammatory cytokines (IL-1β and IL-22) is connected with migration and invasion through BRG1 downregulation of the Wnt/β-catenin signaling pathway and is responsible for cancer growth and invasiveness.

Data point to different expression profiles of miRNAs for normal, inflamed, or cancer tissues with overexpression of miRNAs (miR-215-5p, and miR-3065-5p) seen in inflamed tissue. These individual miRNAs differ between inflamed or normal tissue and cancer tissue. They are specified even in the early stage of tumors and participate in creating tumor microenvironments. Higher expression of miRNAs is observed in inflamed tissue and is supposed to be a potential regulator of chronic inflammation and carcinogenesis (miR-21-5p and miR-30b-5p). Macrophages stimulated with IL-4 influence oncogenic miRNAs (miR-223) linked to the invasiveness of breast cancer cells. Identified specific miRNAs are associated with clinical stage I to IV of cancer and linked to the pathogenesis process, unique for migration (miR-200, miR-10b, and miR-21) and invasion processes (miR-16, miR-92, and miR-494).

HIF-1α plays a significant role in immunity pathways linked to inflammation and cancer pathogenesis. An increased expression of HIF-1α is connected with the progression of different cancers, promotion of angiogenesis, neovascularization, and sustained inflammation [116]. HIF-1α is regulated by pro-inflammatory cytokines (IL-1b, and TNF-α,) in an NF-kB-dependent manner. The level of protein is controlled for example by TARBP2, which prevents its proteasomal ubiquitination to enhance HIF-1α protein stability [117]. A significant correlation of these proteins is observed with breast cancer pathogenesis, and progression process, and is linked to hypoxia.

### 2.3. miRNA and Estrogens

There is an additional link between obesity and breast cancer. It is an increased level of estrogens produced either locally within breast adipose tissue or circulating in the peripheral blood [118,119]. Estrogens are one of the most significant risk factors for breast cancer, especially for estrogen receptor-positive breast cancer (ER+BC). High levels of estrogens as it is seen in the case of obesity, expose breast epithelial cells to pro-proliferative and mutagenic action. Increased proliferation rate leads to the accumulation of mutations and additionally is connected with higher mitochondrial activity and elevated production of reactive oxygen species (ROS) [120]. ROS are generated also during the intracellular metabolism of estrogen, which leads to the production of quinones. Subsequently, quinones form adducts with DNA, which leads to DNA damage. Because of the tumorigenic potential of estrogen, early menstruation and late menopause increase the risk of breast cancer [121].

In premenopausal women, estrogens are synthesized primarily in the ovarian follicles and corpus luteum. After menopause, estrogens are produced mainly in adipose tissue, where they act locally or may be secreted into the bloodstream [122]. However, in adipose tissue, de novo synthetization of gonadosteroids does not occur, only conversion of estrogens precursors: androstenedione, dehydroepiandrosterone (DHEA), and DHEA sulfate (DHEA-S) [122]. High expression of enzymes responsible for catalysis of C19 steroid aromatase, 17β-hydroxysteroid dehydrogenases (17βHSDs), and CYP1B1 is observed in adipose tissue stromal cells and preadipocytes. Tissue levels of estrogens correlate with BMI and the strongest association is observed after menopause [123,124].

The key enzyme of estrogen biosynthesis after menopause is aromatase (CYP19A1, Cytochrome P450 Family 19 Subfamily A Member 1), an enzyme complex found in both breast adipose tissue and tumor tissue. Aromatase is responsible for the conversion of androgens produced by the adrenal cortex, particularly testosterone and androstenedione, resulting in the formation of estradiol (17 β-estradiol, E2) and estrone, respectively. Obesity leads to the induction of aromatase expression in adipose stromal cells (ASCs) and enhances estrogen levels as a result of increased COX-2 expression and elevated levels of proinflammatory mediators and cytokines: TNFα, IL-1, and IL-6 [125,126]. An elevated level of leptin also induces the expression of aromatase by inhibition of p53, which acts as a repressor for aromatase expression [127].

**Figure 3:**
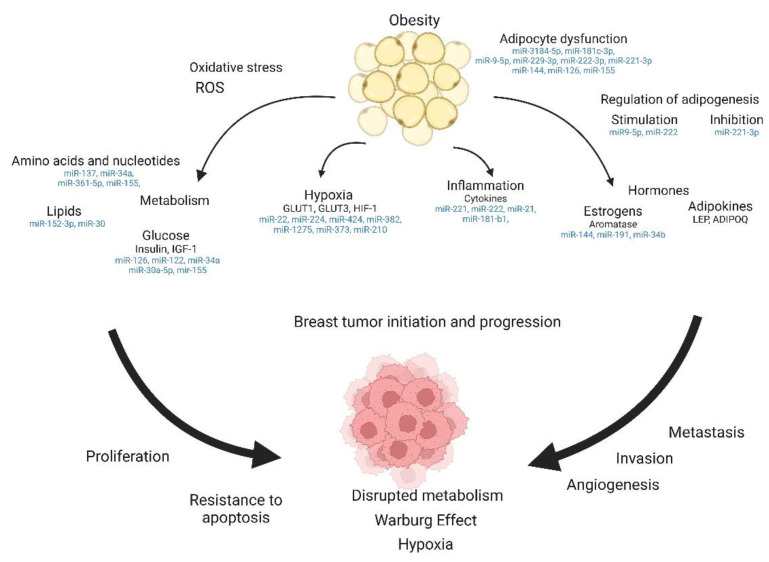
Obesity-related breast tumor initiation. The above figure was created with BioRender.com. Agreement number: KP24PM3RRF.

As the main estrogen, 17 β-estradiol plays a pivotal role in the regulation of the development and growth process of both normal and malignant mammary tissues. E2 exerts a biological effect on cells through genomic action (binding and activating estrogen receptors ERα and Erβ, which bind with ERE) and non-genomic action via binding to G protein-coupled estrogen receptor 1 (GPER1) [Figure 3]

In the context of cancerogenesis, estrogenic activation of GPER1 signaling is considered a factor inducing oncogenic effects [128], while activation of genomic receptors can suppress oncogenes expression. However, the impact of the genomic action of E2 depends on the type of estrogen receptor bound with E2 since activation of ERα and ERβ leads to induction of transcription of different subsets of genes. Cancer growth and development are mainly promoted by activation of ERα [129], whereas the ERβ receptor, as has been shown both in a murine model in vivo [130] and in vitro studies, acts as an anti-tumoral agent [109,110]. Ultimately, the effect of estrogenic activation in mammary epithelial cells depends on the ratio of both receptor types: ERα and ERβ [111].

GPER1 is expressed in normal mammary epithelium and up to 60% of breast cancer tissues [149,150], either in ER-positive or TNBC. The oncogenic potential of signaling in estrogenic activation of GPER1 leads to the induction of proliferation. These effects of E2-GPER1 activation are partially a result of changes in miRNAs expression profiles. Vivacqua A. et al. described the activation of the E2-GPER1-PI3K-Akt-ERK1-Elk1 axis in ER-negative breast cancer cells, which leads to over-expression of oncomiR-144 that in turn targets tumor suppressor transcription factor (Runx-1) and, consequently, causes the increment of cell proliferation [151]. However, opposite results have been shown when presenting the anti-tumoral potential of GPER1 in TNBC. The impact of GPER1 activation in TNBC is widely described by Treeck O. and coworkers [152].

Disturbed expression of ERs in epithelial breast cells causes changes in signaling pathways and may lead to cancer initiation. During cancer progression, ERs expression is modulated both in breast cancer cells and in cells of tumor microenvironment like CAFs or adipocytes. In normal breast tissue, the expression of ERα is high but limited to about 10% of lobular and ductal epithelial cells, while ERβ is expressed in more than 70% of these cells [153].

The normal phenotype of mammary epithelial cells depends on the low ratio of ERα to ERβ expression. However, the expression ratio between both ERs isoforms is dynamic, and neoplastic transformation changes this ratio into high ERα and low ERβ expression levels [153]. ERs expression shift leads to a change of estrogen signaling from normal to tumorigenic. A decrease in ERβ expression is correlated with the malignant process and ERβ has been detected only in 20–30% of metastatic breast cancer with a lack of expression in some invasive breast tumors [154]. Overexpression of ERα, in estrogen receptor-positive BC, is observed in nearly 70% of breast cancer cases. Oncogenic pathways induced by ERα are often mediated by the interaction between miRNAs and targeted oncogenes or tumor suppressors. Estradiol-activated ERα elevates oncomiRs expression, which leads to a decreased expression of tumor suppressors gens. Such a mechanism was demonstrated by Tian X. and Zhang Z. In detail, estrogen-induced miR-191 targeted and decreased the expression of DAB2 and led to the promotion of cellular proliferation of ER+BC cells in vitro. Thus, it has been shown that activation of miR-191/DAB2 axis is crucial in estrogen-induced ER+ breast cancer growth [155]. The oncogenic action of estrogen in breast cancer is also a result of the inhibition of tumor suppressors miRNAs. It was shown that estrogenic activation downregulates miR-34b in MCF-7 cells. The binding of E2 to the ER-p53 complex blocks its transcription activity and decreases miR-34 expression. As a result, expression of miR-34b targets genes leading to an increase of cyclin D1 and Jagged-1 (JAG1) and subsequent induction of cell proliferation. However, ERα-E2 may also exert an antitumor effect as it is indicated by the activation of the expression of tumor suppressor miR-1271, which targets SNAI2 and suppresses the process of epithelial-mesenchymal transition (EMT) in breast cancer cells. The antagonizing role of ERα in the signaling of the EMT process is, among others, one of the reasons why the depletion of ERα induces metastasis of breast cancer [156]. During breast cancer progression from estrogen-dependent to a more invasive tumor, ERα expression is decreasing until an estrogen-independent phenotype occurs, which correlates with a more aggressive phenotype and poor clinical outcome. In the context of therapy, loosing of ERα expression in breast cancer cells causes drug resistance to endocrine therapies based on targeting the ERα like SERM (selective estrogen receptor modulators) or SERD (selective estrogen receptor down regulators). SERMs are competitive ERα antagonists like tamoxifen, which binds and blocks ERα. Fulvestrant belongs to SERDs, newer ERα antagonists, which lead to the degradation of the ERα. One of the factors causing changes in ERs expression levels is the alteration of microRNA expression. Examples of miRNAs that decrease ERs expression in ER+BC directly by binding to 3′UTRs of ER transcripts are miR-142-3p or miR-21 [157,158]. The indirect influence of miRNAs on ER expression can be obtained by targeting transcripts coding proteins involved in epigenetic regulation of ER expression as DNA methyltransferase 1 (DNMT1). Xu Y. et al. showed, in two breast cancer cell lines, HCC1937 and MCF7, that miR-148a decreased DNMT1 expression, which resulted in the demethylation of the ER-α gene, which in turn led to an increase of ERα expression [159]. Additionally, the activity of ERs depends on the level of E2. For this reason, miRNAs involved in the regulation of expression of E2 biosynthesis pathway proteins should also be considered. As mentioned above, the key enzyme of estrogen synthesis is aromatase, which catalyzes reactions leading to the formation of estrogens from other steroids [160]. Aromatase is the next target in endocrine therapy of ER+BC and is based on drugs that inhibit CYP19A1 activity, aromatase inhibitors (AIs). AIs lead to the reduction of estrogen production. A recent study has shown that miR-19a-3p, which decreases CYP19A1 expression, was overexpressed in breast cancer plasma patients and reduced the sensitivity of ER+ breast cancer cells to one of the AIs, letrozole [161]. An additional layer of the complex regulation of ER expression is the interplay between miRNAs and E2-ERs–signaling in breast cancer. Induced by E2, direct interaction of ERα with the promoter region of miR-196a leads to upregulation of miR-196a expression [162]. Based on the microarray study, Bhat-Nakshatri et al., in 2009, identified the miRNAs whose expression was regulated by estradiol in MCF-7 breast cancer cells. According to these results, E2-ERs repressed the expression of 7 miRNAs while inducing the expression of 21 miRNAs including Let-7 family members, miR-98, and miR-21. However, only the activated complex of ERα–E2 upregulated miR-21 expression because unliganded ERα led to suppression of miR-21 expression [163]. Results presented by Yang H. and co-workers are important examples of cross-talk between miRNAs and ER signaling, as in their study, E2 decreased the expression of miR-124, while increasing GPER1 expression in MCF-7 cells. GPER-E2 signaling suppressed miR-124 and led to overexpression of CD151, and in addition, promoted proliferation, invasion, and migration of breast cancer cells [137]. This interplay between ERα, GPER1, and miRNAs is an important element of resistance to anti-estrogen therapy since it has been demonstrated that antagonists of the Erα, such as tamoxifen or fulvestrant, may activate GPER1 [164]. Moreover, many studies have shown a possible association of GPER1 expression with the development of tamoxifen resistance [165,166,167,168,169].

As mentioned above, miRNA regulation of estrogen signaling through changes in GPER1 or ERs expression level is a very important mechanism, especially in the context of endocrine therapy or drug resistance. miRNAs involved in shifting of ERα and ERβ expression during cancer progression lead to increased expression of ERα in early stages and decrease ER expression in advanced breast cancer. Inhibition of oncomiRs or induction of miRNA suppressors expression could lead to the restoration of ER expression in advanced stages of breast cancer and increased sensitivity toward SERMs or SERDs. Examples of oncomiRs mentioned above, miR-142-3p and miR-21, which decrease ERα expression in ER-positive breast cancer, may contribute to tamoxifen resistance [96,97]. Other examples of miRNAs targeting ERα and associated with the responsiveness of breast cancer patients to SERMs could be miR-221/222, miR-18a, miR-19a/b [170], and miR-182-5p, targeting FOXO3a [171] and miR-575 regulated by ERα [172].

Interesting results presented by Zhou et al. demonstrated the difference in miRNAs expression profiles between MCF-7 cells resistant to tamoxifen and MCF-7 cells resistant to fulvestrant. Among differentially expressed miRNAs, miR-342-5p (associated with ERα and BRCA1 expression in breast cancer) and miR-1226 were downregulated in tamoxifen-resistant cells. Fulvestrant resistance was associated with overexpression of miR-3188 and miR-21 as well as downregulation of suppressor miR-149. Moreover, miR-4532, miR-486-5p, and miR-138, among others, are common miRNAs dysregulated in cells resistant to both tamoxifen and fulvestrant and could serve as markers for drug resistance [173].

Searching for miRNAs being involved in a patient’s response to therapy is very important for the understanding of drug resistance mechanisms and may lead to finding new targets or creating personalized therapy for breast cancer.

## 3. Clinical Significance—Diagnosis and Therapy

Disturbed miRNA expression profiles are identified in the tissues and serum of breast cancer patients. Therefore, these profiles may point to miRNAs that could be used as new clinical biomarkers or targets in breast cancer therapy. Many studies indicate the diagnostic potential of individual miRNAs (miR-19b-3p, miR-20b-5p, miR-24-3p, miR-92a-2-5p, miR-106a-3p, miR-106a-5p, miR-152, and miR-210) or combinations of these miRNAs. These molecules (for example miR-1246+miR-206+miR-24+miR-373) allow the detection of breast cancer with high specificity (96%) and sensitivity (98%) even in the early stages of cancer development [174]. Moreover, expressions of miRNAs changes are associated with the subtype of breast cancer (miR-16-5p, miR-21-5p, miR-342, and miR-199a-5p), the clinical stage, and the severity of breast neoplasm (miR- 21, miR-210 and miR-221), or are correlated with the progression of breast cancer (miR-525-5p, and miR-106a-5p).

Depending on the role of miRNA, different expression patterns are identified in the tissue of oncological patients. Decreased levels of unique miRNAs are observed in breast cancer patients. miR-145, miR-125a, and miR-206 show lower expression in breast cancer tissue compared to adjacent normal tissues. The level of miRNAs is negatively correlated with tumor size, lymph node metastasis, and status of ER and HER-2 [175]. Downregulation of miR-142-3p, an important suppressor of breast cancer oncogenesis [157], is associated with ER-positive breast cancer and predicts poor survival of patients in grade 3 breast cancer [157,176]. The low expression also applies to miR-424-5p and is associated with advanced clinical stage, increased tumor size, lymph node metastasis, distant metastasis, and poor histological grade in basal-like breast cancer patients [177]. Moreover, miR-21, miR-210, and miR-221 also correlate with poorer asymptomatic and overall survival of patients and, thus, play a significant role in triple-negative primary breast cancer.

In contrast, the expression of miR-21 and miR-10b is significantly higher and positively correlated with breast cancer tumor size, lymph node metastasis, expression (miR-10b), and lymph node metastasis in postmenopausal patients [175]. Higher expression of miR-21, miR-210, and miR-221 has also been shown for triple-negative primary breast cancers [178]. The serum miR-21 has also been previously reported as a possibly independent poor prognosis factor in breast cancer patients [179].

Many miRNAs have been found as “major diagnostic microRNAs for early diagnosis of breast cancer” (miR-133b, miR-145-5p, miR-15a-5p, miR-16-5p, miR-181a-5β, miR-18a-5β, miR-21-5β, miR-365a-3β and miR-92a-3β); “major oncogenic microRNAs in breast cancer” (miR-21-5p); “large tumor-suppressing microRNA in breast cancer” (miR-126-5p); “Negative prognostic microRNA signatures in breast cancer” (miR-21-5p) [179,180].

Disturbed metabolism and inflammation related to obesity are regulated with miRNA and linked to a different pattern of miRNA profiles. Hyperglycemia-induced pathways are correlated with elevated expression of miR-467 in tumor tissue, breast cancer cells, and macrophages. miR-467 targets thrombospondin-1 (TSP-1) and regulates the processes of cancer inflammation, promoting inflammation by the recruitment of tumor macrophages and fastening tumor growth connected with high blood glucose influence on angiogenesis processes [27]. The miR-467 level is significantly upregulated in hyperglycemic patients (258-fold compared to normoglycemic patients and 56-fold in adjacent normal tissue) [27]. Additionally, miR-467 downregulates the expression of TSP-1 and increases angiogenesis. The use of the miR-467 antagonist in murine models of hyperglycemia resulted in a reduction of the tumor mass and inhibition of progression, including the angiogenesis process. These findings also confirmed earlier conclusions [181] about regulating the target signaling pathway of glucose metabolism and inflammation [182]. Another oncomiR, miR-155, is involved in disturbed metabolic processes and promotion of breast cancer, and these observations allowed starting to research the use of anti-miRNAs in cancer immunotherapy. miR-155 is a key regulator of glucose metabolism in breast cancer [183] and is linked to prostaglandin metabolism in the tumor. In the two independent large cohorts of breast cancer studies, the miR-155-5p expression was associated with negative prognostic factors, including reduced expression of hormone receptors, high histological grade, and proliferation Ki67 index [184]. Inhibiting of miR-155 with antagomiR (MRG-106) is tested as a cancer therapy (a phase-1 clinical trial) [185] to influence the Cox2/ PTGES1/PGE2 pathway and reducing PGE2 in the course of breast cancer progression.

Above mentioned miRNAs with diagnostic potential are associated with inflammation. They target inflammatory molecules and cytokines (as miR-146a targets IL-17). An elevated level correlates negatively not only with patient survival but also with sensitivity to cancer therapy [186].

Moreover, results of studies and observations suggest that the miRNAs may be classified not only as a novel candidate for diagnostic and prognostic indicators but also as potential therapeutic targets in new treatment strategies, especially in clinically aggressive breast cancer associated with obesity. Furthermore, miRNAs can be potentially used as therapeutics agents as they modulate the function of a gene by influencing its expression and global cellular pathways. Modulation of miRNAs expression, due to the inhibition of oncogenic miRNAs or replacement of deficient tumor-suppressive miRNAs, could be considered a novel treatment technique. Up to date, candidates miR-based therapeutics are in drug development process or clinically tested (phase I and phase II clinical trials) [187].

miR-142-3p, which suppresses expression of the oncogene HMGA1 and targets the AKT/ERK/STAT3 pathway, is supposed to be a new miRNA for breast cancer therapy that restores this suppressive miRNA in tumor breast cells using a targeted nanoparticle delivery system [176,188].

According to progress in endocrine therapy of breast cancer patients, drugs based on targeting ER make an increasingly important strategy of treatment. However, drug resistance significantly limits the effectiveness of this treatment. Poor response to endocrine therapy estimated at 30% of breast cancer patients [189,190] prompts the search for miRNAs correlated with SERMs/SERDs resistance, sensitization, or simply a prognostic biomarker. miR-221 can be considered a potential biomarker of tamoxifen resistance. It was shown in a case-control study that serum miR-221 expression level is higher in tamoxifen-resistance luminal-subtype breast cancer patients with local recurrence and metastasis than in patients without progression [191]. Additionally, the inhibition of miR-221 and miR-222 restored the sensitivity of tamoxifen-resistant breast cancer cell line (MCF-7 TamR) cells to tamoxifen [192]. Inhibition of miR-221/222 also restored the expression of ERα and PTEN, arrested cells in the G1 phase, and finally resulted in reduced cell growth and cell migration [192].

Another example of the crucial role of miRNA in the induction of chemoresistance in breast cancer is miR-9-5p. miR-9-5p is an important factor affecting the prognosis of breast cancer and its high expression levels suggest a poor prognosis for patients [193,194]. The team of Liu indicated that miR-9-5p influences tamoxifen resistance in the MCF-7 cells [65]. Additionally, it was described that an increased ADIPOQ expression resulting in autophagic death of breast cancer cells is associated with a higher survival rate of breast cancer patients treated with chemotherapy [64]. Another study presented miRNA signature related to resistance to neoadjuvant chemotherapy. The experiment was based on the comparison of miRNA expressing profiles between 10 addressed miRNAs. The results showed that miR-23a-3p, miR-200c-3p, and miR-214-3p were upregulated, while miR-451a and miR-638 were downregulated. These particular miRNAs target genes involved in signaling pathways of mTOR, Wnt, and ErbB and processes related to p53 regulation, ubiquitin-mediated proteolysis, and cell skeletal protein regulation [195]. Identification of miRNAs signature potentially allows predicting a patient’s response to chemotherapy and to choose the best chemotherapeutic regimens for breast cancer patients.

## 4. Conclusions

Understanding the interaction and processes that link inflammation, obesity, and the risk of cancer is crucial not only for understanding the pathogenesis of breast cancer but, from the clinical point of view, for developing new biomarkers and targets for efficient therapies for patients. Research conducted over the past decade on the distribution of miRNA expression profiles in breast cancer and the search for a correlation with clinical outcomes as well as molecular targets of miRNAs have allowed the identification of miRNAs signatures linked to pivotal breast cancer processes like tumor size, lymph node metastasis, and status of hormones receptors as well as to inflammation and obesity.

## Data Availability

Not applicable.

## Abbreviations

BC	breast cancer
miRNAs	microRNAs
Her2	human epidermal growth factor receptor 2
TNBC	triple-negative breast cancer
BMI	body mass index
WHR	waist-hip ratio
AT	adipose tissue
IGF-1	insulin-like growth factor
TBARS	thiobarbituric reactive acid substances
MDA	malondialdehyde
LOOH	lipid hydroperoxides
AMPK	AMP-activated protein kinase
ERK	signal-regulated kinase
JAK/STAT	signaling pathways, Janus kinase
PI3K	phosphatidylinositol 3-kinase
MAPK	mitogen-activated protein kinase
MA	mature adipocytes
FOXP4	forkhead box protein P4 ()
PPARα	peroxisome proliferator-activated receptor alpha
MSCs	marrow stromal stem cells
ADIPOQ	adiponectin
ADIPOR2	adiponectin receptor 2
BECN1	beclin 1
GLUT4	glucose transporter 4
SCD1	stearoyl-CoA desaturase-1
DGAT1/2	diacyl glycerol acyl transferase1/2
FAS	fatty acid synthase
AdipoQ	adiponectin
ATGL	adipocyte triglyceride lipase
AP2	adipocyte fatty acid binding protein
IRS1	insulin receptor substrate 1
phosphor-AMPKα	Protein Kinase AMP-Activated Catalytic Subunit Alpha 1
ATM	adipose tissue macrophages
GLUTs	glucose transporters
EVs	extracellular vesicles
TDO2	tryptophan-2,3-dioxygenase
LA	linoleic acid
DHA	docosahexaenoic acid
HIF-1α	hypoxia-inducible factor
VEGF	vascular endothelial growth factor
ADM	adrenomedullin
TGF-β3	transforming growth factor-β3
HK1, HK2	hexokinase
IGF2	insulin-like growth factor 2
C-MYC	myelocytomatosis virus oncogene cellular homolog
TGFβ	transforming growth factor-β
GM-CSF	granulocyte-macrophage colony-stimulating factor
NF-kB	nuclear factor kappa B
STAT3	signal transducer and activator of transcription 3
TLR	toll-like receptor
CAFs	cancer-associated fibroblasts
TAMs	tumor-associated macrophages
ROS	reactive oxygen species
DHEA	dehydroepiandrosterone
DHEA-S	DHEA sulfate ()
17βHSDs	17β-hydroxysteroid dehydrogenases
CYP19A1	Cytochrome P450 Family 19 Subfamily A Member 1
ASCs	adipose stromal cells
GPER1G	protein-coupled estrogen receptor 1
EMT	epithelial-mesenchymal transition
SERM	selective estrogen receptor modulators
SERD	selective estrogen receptor down regulators).
DNMT1	DNA methyltransferase 1
Ais	aromatase inhibitors
